# Introducing genetic testing with case finding for familial hypercholesterolaemia in primary care: qualitative study of patient and health professional experience

**DOI:** 10.3399/BJGP.2021.0558

**Published:** 2022-06-14

**Authors:** Luisa Silva, Laura Condon, Nadeem Qureshi, Brittany Dutton, Stephen Weng, Joe Kai

**Affiliations:** Centre for Academic Primary Care, University of Nottingham, Nottingham.; Centre for Academic Primary Care, University of Nottingham, Nottingham.; Centre for Academic Primary Care, University of Nottingham, Nottingham.; Centre for Academic Primary Care, University of Nottingham, Nottingham.; Centre for Academic Primary Care, University of Nottingham, Nottingham.; Centre for Academic Primary Care, University of Nottingham, Nottingham.

**Keywords:** cholesterol, familial hypercholesterolaemia, genetic testing, genomic testing, primary care, qualitative research

## Abstract

**Background:**

Familial hypercholesterolaemia (FH) is a common inherited condition causing elevated cholesterol, premature heart disease, and early death. Although FH can be effectively treated, over 80% of people with FH remain undetected.

**Aim:**

To explore patient and health professional experiences of introducing genetic testing with case finding for FH in primary care.

**Design and setting:**

Qualitative study in UK general practice.

**Method:**

Semi-structured interviews with a purposeful sample of 41 participants (24 patients and 17 health professionals) from eight practices, using an electronic case-finding tool (FAMCAT) to identify patients with higher likelihood of having FH and who were then offered diagnostic genetic testing in primary care. Data were analysed thematically.

**Results:**

While prior awareness of FH was low, patients were unsurprised to be identified as being at risk, and positive about being offered genetic testing by their practice. Patients not found to have FH were relieved, although some felt frustrated that their high cholesterol lacked a clear cause. Those confirmed to have FH largely expected and accepted this outcome. Practitioners saw detection of FH as an important new opportunity for preventive care. They found the case-finding tool easy to apply and noted patients’ high uptake of genetic testing. While they were comfortable referring appropriate patients for further specialist management, GPs sought clearer definition about responsibility for identification and long- term care of FH in future care pathways.

**Conclusion:**

Introducing genetic testing with electronic case finding for FH in primary care was positively experienced by patients and practitioners. Further development of this approach could help improve detection of FH in the general population.

## INTRODUCTION

Familial hypercholesterolaemia (FH) is one of the most common inherited conditions, causing elevated total cholesterol (TC) and low-density lipoprotein cholesterol (LDL- C) from birth, and a 13-fold greater risk of premature coronary heart disease (CHD) and early death.^1^ Diagnosis to enable effective high-potency lipid-lowering treatment and lifestyle advice can ensure that patients with FH have healthy lives and normal life expectancy.^2^ However, over 80% of people with FH have not yet been diagnosed, with around 234 000 people still unidentified in the UK.^2^ In addition, the identification of index cases can enable ‘cascade’ testing, as 50% of first-degree relatives will also be affected by the condition.

National Institute for Health and Care Excellence guidance in the UK^3^ recommends that general practices search health records for people with TC >9.0 mmol/L if aged ≥30 years, or >7.5 mmol/L if aged <30 years, as they are at highest risk of having FH. Possible FH may also be indicated by the Simon Broome criteria of TC >7.5 mmol/L or LDL-C >4.9 mmol/L in those who also have a family history of premature myocardial infarction (aged <60 years in a first-degree relative).^3^^,^^4^ It is recommended that these patients are referred to specialist lipid services for diagnostic genetic testing, further management, and testing of relatives if appropriate. However, most patients referred to lipid specialists do not transpire to have FH, while others do not attend after referral.^5^

The potential of exploiting genomics and identifying FH in primary care is increasingly recognised.^6^^–^8 In the UK, a predictive clinical case-finding tool to identify patients at highest likelihood of having FH (familial hypercholesterolaemia case ascertainment tool [FAMCAT]) has been developed^9^ and validated^10^ for use with patients’ routine electronic health data in primary care. Readier access to genetic testing in the community, when the possibility of FH is identified by their GP, could facilitate targeted referral for lipid specialist assessment, and enable more patients with undetected FH in the general population to be diagnosed. As part of a wider evaluation of introducing FAMCAT and genetic testing for FH in primary care,^11^ this qualitative study explored participating practitioners’ and patients’ experiences of this approach.

## METHOD

### Context

The wider study involved 14 general practices applying a validated electronic FH case ascertainment tool (FAMCAT) to their practice list.^11^ The FAMCAT algorithm includes elements of existing clinical criteria for FH, such as the Simon Broome criteria and searching variables available from patients’ electronic health records, to identify those with highest likelihood of FH. It takes account of interactions between statin prescribing, family history, total cholesterol, triglycerides, LDL-C, and secondary causes of elevated lipids such as diabetes and chronic kidney disease.^10^ It is intended as a case-finding tool to identify those eligible for further assessment and testing for possible FH.

**Table:** How this fits in

Over 80% of patients with familial hypercholesterolaemia (FH) remain unidentified, with diagnostic testing occurring in specialist lipid care. This study has found that introducing electronic case finding and genetic testing for FH into primary care was positively experienced by patients identified at high risk, and positively perceived by practitioners as important, straightforward, and similar to existing preventive care. With further development and evaluation, this offers a promising approach to help improve detection of FH in the general population.

A cohort of 336 patients identified by FAMCAT as being at higher risk of having FH, who consented to study participation, were posted information about possible FH and invited to have blood tests for current cholesterol and genetic testing for FH. Participants also completed a family history questionnaire for the wider study.^11^^,^^13^ Genetic testing was completed by 283 patients. This followed practices’ usual procedures for venesection at their surgery or local community phlebotomy clinic. On average, patients waited 3 months between having their blood test and receiving results by letter. Further detail on the testing used is provided in Box 1. There were four possible results, summarised in Table 1.

**Box 1.** Genetic testing for FH

Next-generation sequencing (NGS) was used and is now becoming more available in the UK. This technique identifies a genetic mutation causing monogenic FH (caused by a major change in a single gene). The NGS assay identifies FH-causing genes *LDLR* (18 exons), *APOB* (28 exons), *PCSK9* (12 exons)*,* and *LDLRAP1* (9 exons). It further identifies genetic variations called ‘variants of unknown significance’ (VUS) that may indicate the person has FH, or simply reflect normal variation. The assay also identifies 12-point mutations (LDL-C raising single nucleotide polymorphisms). Each of these slightly increases cholesterol levels. If several are present collectively they contribute to ‘polygenic hypercholesterolaemia’. This means the patient may be more prone to having raised cholesterol than the general population, but does not have FH.^12^

*FH = familial hypercholesterolaemia. LDL-C = low-density lipoprotein cholesterol.*

**Table 1.** Genetic test results

**Test result**	**Advice to GP**	**Advice to patient**
**FH mutation positive**	Confirms patient has FH. Refer to lipid specialist	Patient informed has FH. Will be referred to lipid specialist
**FH mutation unclear (VUS)**	Result unclear. Patient may have FH. Refer to lipid specialist for further assessment	Result unclear. May have FH. Require specialist referral and assessment
**FH mutation negative**	Patient does not have FH. Provide healthy lifestyle advice and leaflet. However, if positive family history of premature heart disease, the patient fulfils Simon Broome criteria for possible FH, as per NICE guidelines. Seek specialist advice on whether further assessment needed	Informed does not have FH. If any relative develops heart attack aged <60 years, may need specialist assessment, advised to see GP
**FH mutation negative with polygenic result**	Patient does not have FH but genetic testing indicates more likely to have raised cholesterol than general population (Box 1). Advise regular routine cardiovascular risk assessment	Informed does not have FH but is more prone to high cholesterol. Advised to see GP for routine cardiovascular health check

*FH = familial hypercholesterolaemia. NICE = National Institute for Health and Care Excellence. VUS = variant of unknown significance.*

### Sampling and data generation

A purposeful sample was selected from all patients identified with possible FH who were offered testing and willing to participate in the study, to reflect experience of differing test outcomes and a diversity of family history and social and educational background (an estimated 20–30 patients required). Sampling of healthcare professionals included a range of roles (clinical, administrative, and managerial), practice population demographic and locality (inner city, suburban, and rural), and clinical experiences of FH in relation to patient identification using FAMCAT, genetic testing, referral, and management (an estimated 15–20 professionals required). Professionals were interviewed after they had had at least 6 months’ experience applying FAMCAT and implementing genetic testing in their practice. One-to-one semi- structured interviews using topic guides with broad prompts (see Supplementary Appendix S1) exploring patient and professional experiences were conducted by two post-doctoral researchers not involved in the development of the FAMCAT tool, either face-to-face or by telephone according to participant preference. Before the interviews all participants provided written informed consent.

### Data analysis

Interviews were digitally audiorecorded and transcribed verbatim. Data were analysed thematically^14^ and concurrently, with sampling and data generation continuing until saturation of themes was reached. Analysis followed a process of familiarisation with data, generation of initial codes, searching for and reviewing themes, and defining and naming themes.^14^ Data were manually reviewed and emerging themes developed by the researchers undertaking the interviews and a third senior researcher, who had differing disciplinary backgrounds in health psychology, health services research, and general practice. The senior researcher was involved in the prior development of the FAMCAT tool, but not in its piloting in participating practices. All responders were sent a draft summary of findings to check^15^ and any comments on the interpretation were invited. Responses from 11 interviewees were received confirming study findings as true to their experiences with no further comments made. These steps helped to further refine the analysis and check the interpretation, as a clear descriptive narrative reporting participants’ experience in relation to possible FH identification and genetic testing. In addition to the latter ‘member checking’, interim comment was sought from the study patient and public involvement (PPI) advisor (who has FH) on the first seven patient interview transcripts and their preliminary analysis; and emerging themes were further discussed with three FH specialist health practitioners providing the patient telephone helpline of the national FH charity, Heart UK.

## RESULTS

### Participant characteristics

Forty-one participants were purposely selected and interviewed. These included 24 patients with a range of FH test outcomes, family histories, and socially diverse backgrounds, and 17 primary care professional responders (Table 2). Health professional responders (10 female, 7 male) came from inner city (*n* = 3), suburban (*n* = 3), and rural (*n* = 2) practices, with socially diverse populations and practice Index of Multiple Deprivation (IMD) scores ranging from 6.3–48.8 (data not shown).^16^ GPs interviewed had been qualified for an average of 22 years (range 13–36 years).

**Table 2.** Characteristics of participants (*N* = 41)

**Participants**	***n***
Patient FH mutation positive	8
Patient FH mutation unclear (VUS)	3
Patient FH mutation negative	7
Patient FH mutation negative, prone to high cholesterol (polygenic)	6
GP	7
Practice nurse	4
Healthcare assistant	4
Practice manager	2

**Patients’ sex**	
Male	11
Female	13

**Patients’ age, years**	
<40	1
40–49	7
50–59	8
>59	8

**Patients’ ethnicity**	
White British/Irish	16
South Asian	4
Black	1
Other European	3

**Patients’ IMD^a^**	
Quintile 1	7
Quintile 2	5
Quintile 3	7
Quintile 4	2
Quintile 5	3

**Patients’ highest formal educational level**	
No formal qualifications	4
GCSE/O-level/CSE	5
A-level or equivalent	6
Degree	4
Other/vocational	5

**Family history — first-degree relatives**	
Known FH	0
High cholesterol	17
Premature CHD events (aged <60 years)	8
Premature deaths from CHD (aged <60 years)	4

**Comorbidities (apart from elevated cholesterol)**	
None	14
CVD	3
Other (for example, lymphoma, asthma, hypothyroidism, hypertension, and diabetes)	7

a *IMD quintile where 1 is least deprived and 5 is most deprived. CHD = coronary heart disease. CVD = cardiovascular disease. FH = familial hypercholesterolaemia. IMD = Index of Multiple Deprivation. VUS = variant of unknown significance.*

### Findings

The main themes generated from the data reflected the experiences and perspectives of patient and healthcare professionals during the process of case finding and genetic testing. They included identification of higher risk of FH, experience of testing offer, testing process, experience of FH test results, and experience after specialist assessment.

#### Identification of higher risk of FH and experience of testing offer

Both patients and GPs positively anticipated the value of improving identification of FH, recognising potential benefits for patients and their families’ long-term health. Patients’ motivations for having FH testing were to detect an asymptomatic condition, so they could manage it appropriately and enable other (especially younger) members of their family to be tested if needed:

*‘This is excellent because it is primary prevention and we’re hoping that would have a better effect for secondary care in the future with less toll on the system if we identify these patients early so they don’t have to go into hospital with a massive MI* [myocardial infarction] .*’*

(GP6)

*‘The main reason I wanted that* [test] *to be done is that my children could have that sort of genetic, I was worried for myself but I thought … it would be helpful to them to know if that was the case and then they are being made aware of it.’*

(Patient participant [PP]4, 58 years old, subsequent FH mutation negative)

Primary care practitioners found that the electronic search to identify people at greater risk of having FH was uncomplicated to implement on practice IT systems. Having search criteria for clinical variables already incorporated in the FAMCAT tool rather than needing to input a number of Read codes themselves aided the process and identification of relevant patients. (Read codes are a coded thesaurus of clinical terms that provide a standard vocabulary for clinicians to record patient findings and procedures in health and social care IT systems across primary and secondary care.) For example:

*‘*[…] *to run this* [FAMCAT] *search and then to come out with a list of, “right this is what you’re doing”, and it was really straightforward. It was off the back of this complicated report that had been built already rather than just me trying to make sure that I’ve included all the Read codes recorded in the clinical systems* […] *that was definitely a real bonus.’*

(Practice nurse [PN]3)

Practitioners reflected that seeking patients at higher risk of FH and offering relevant blood testing was similar to other screening they already routinely undertook for other conditions in general practice, such as diabetes. For example:

*‘*[…] *it doesn’t seem particularly different to other areas of screening that we are involved in and it is for preventative health.’*

(GP2)

*‘Yes definitely, it’s been the same* [process] *for diabetes and lots of other* conditions.’

(GP9)

Very few patient participants were previously aware of FH as a condition. However, most were unsurprised that their practice had invited them to be tested. Receiving information about possible FH with an invitation for having blood testing was regarded as straightforward. Patients understood this, given their personal history of elevated cholesterol, or family history of FH or heart disease, and felt familiar given their prior experience of cholesterol monitoring and review. For example:

*‘To be honest when I received the* [invitation for testing] *letter I wasn’t overly surprised because quite a lot of people in my family have high cholesterol, so I wasn’t surprised to have been identified.’*

(PP21, 40 years old, subsequent FH mutation negative with ‘polygenic result/prone to high cholesterol’)

*‘I had never heard of it* [FH] *before but obviously we have a lot of heart trouble and everything in the family, it felt something that I could take part in … ’*

(PP7, 60 years old, subsequent FH mutation negative)

*‘Well at the time I certainly didn’t know anything about it* [FH]*, obviously when I received the letter I thought, because I have had such high cholesterol levels in the past, I thought maybe I ought to go for this test and just see really, but at the time I didn’t really know anything about it.’*

(PP17, 59 years old, FH mutation positive)

Patients appreciated that testing of their relatives might be needed depending on the outcome of their own test. Many discussed their invitation for testing with partners, siblings, children, and sometimes their own parents to ask about family history. While this had not caused concern for most, some anxiety about possible family implications could arise:

*‘As soon as I did mention it* [testing] *to my daughter in law … she said to me, “oh would it be passed on to the children?” meaning the grandchildren, and I said, “well nothing has come back* [to say] *I have actually got it so until that does, I wouldn’t worry yourself about it”. So they were a bit … a few alarm bells going off … ’*

(PP4, 58 years old, subsequent FH mutation negative)

There was some initial scepticism among practitioners about likely patient engagement among some responders, particularly in inner-city practices with previous experience of low response rates to invitations for preventive health checks. However, this was confounded by positive experience of generally high uptake of testing among patients invited, which was attributed to overall awareness of cholesterol as a health issue and existing lipid-lowering treatment:

*‘Sometimes you just do it* [arranging appointments for blood testing] *and you don’t actually think that anyone will show up do you? But yes, it is good that it* [attendance] *shows that it is working. And that is something that we enjoy as well … High cholesterol … has been in the news and papers a lot more hasn’t it and dietary advice is changing isn’t it.’*

(PN2)

*‘“When will I get my results?” they were quite looking forward to the process so they* [the patients] *seemed to be more engaged.’*

(PN1)

#### Testing process

Patients felt that the written information on FH accompanying their invitation for testing was adequate, and did not feel the need to discuss this further with their GP before having blood testing. However, the considerable time they waited to receive results was noted and this caused a degree of concern for some responders:

*‘I did think it was a long time* [waiting for results] *… A little bit anxious but not too bad, obviously I wanted the result but yes, I wasn’t too bad no.’*

(PP17, 59 years old, subsequent FH mutation positive)

Communicating general information about possible FH and genetic testing to patients was not perceived to further impact on practice resources with patients routinely attending for testing following their invitation. Although offering genetic testing for FH was viewed as a positive development for patient care with simple blood testing, there was some concern its organisation could encroach on practice staff time and cause inefficiencies within their existing workload. Testing required scheduling of appointments for blood samples so that they could be sent to the laboratory within 48 hours of collection:

*‘Anything that is time or cost neutral for the practice and has a clear benefit for people we’ll generally say yes, we will go for it and if it can be really clear that that is the case then we go for it big time* [but] *our staff are quite overloaded with work* […] *’*

(Practice manager [PM]1)

*‘… the taking of the bloods is not a problem, it is just the practicalities.’*

(PM2)

#### Experience of FH test results

Most patients who received a result letter indicating that they did not have FH were relieved for themselves and their family, and were untroubled. However, some felt surprised or perplexed that they were not shown to have FH given their family histories of cardiovascular disease (CVD), and had hoped for an explanation for their pattern of high cholesterol:

*‘I didn’t really feel anything* [when receiving result]. *I was either going to have the gene, a faulty gene or not and I happened to not — so it didn’t really make me feel anything particularly.’*

(PP13, 41 years old, FH mutation negative]

*‘I honestly thought I had got this FH but the letter said that I didn’t so* […] *I was a bit confused* […] *I was really expecting it to say, “yes you have got it”* […] *because* [family members] *they have all had heart attacks, strokes* […] *and I thought well there has got to be something in the gene* […] *so I was quite surprised.’*

(PP23, 66 years old, FH mutation negative, prone to high cholesterol]

Two responders expressed more negative emotions that they had not inherited FH, for example, feeling at fault for developing hypercholesterolaemia:

‘If you had told me it was because of my mother, I would have been happier now, I would have thought, “well there is nothing I can do about that, I have inherited this and I ought to tell my daughter” … but actually when the letter came back saying that it was negative I thought well, that just must mean that it was my fault, so I must admit [that] has made me more miserable.’

(PP3, 58 years old, FH mutation negative)

Mailed results confirming FH or identifying possible FH, both with need for specialist referral, were accepted and largely expected by those receiving this result (one-third of the purposeful sample). Confirmation of FH was perceived to have had modest impact, noting their significant personal and family history of CVD, prior awareness of their elevated cholesterol, and that they were already using lipid-lowering treatment to tackle the problem:

*‘It has not made a lot of difference to me because I was already having my cholesterol* [treatments] *before and tried to bring it down.’*

(PP16, 56 years old, FH mutation positive)

*‘I think I probably knew … that I had that gene. I kept thinking because of my history and then my family history it made me realise that it wasn’t just … there is something there … and obviously the wee bit more was the gene.’*

(PP11, 69 years old, FH mutation positive)

Most practitioners felt that communicating information about possible FH, with invitation for genetic testing for the condition, was realistic. However, the ongoing clinical management of those awaiting the results of their genetic testing for FH was queried by some:

*‘* […] *given that there is then quite a gap until they get the genetic results, are we supposed to treat them in the meantime? As we would do normally by looking at their other risk factors* […] *or do we just sort of wait, put things on hold until they have got the genetic result and then may need referral to the lipid clinic?’*

(GP4)

GPs were comfortable referring patients with results suggesting FH or indicating a variant of unknown significance (VUS) for specialist assessment, but sought greater understanding about interpreting and communicating the range of possible test results, and more in-depth guidance on long-term care of FH. This included the importance of treating elevated cholesterol more aggressively, and what lipid specialists may do beyond prescribing lipid-lowering therapy:

*‘It would be useful to know what further assessment are we talking about here at the hospital, what is it they would do for a VUS? So we need to know a reason why we are referring them.’*

(GP8)

As approaches to improving identification of FH developed, GPs also anticipated a need for clearer guidance about evolving roles at the primary–secondary care interface. In particular, guidance on who may have what clinical responsibilities or duty of care related to genetic testing for FH, and communicating and acting on results appropriately.

*‘If you have done a test you’re responsible for the result … and that is a duty of care and it does fall back to responsibility … and following up on a result that you’ve actioned.’*

(GP7)

#### Experience after specialist assessment

Patients referred for specialist assessment with a confirmed FH mutation felt informed and reassured after being prescribed new medications with subsequent improvement in their cholesterol:

*‘I have been on medication for cholesterol for some years and have struggled to get the cholesterol level below … but then after attending the clinic I was prescribed some different medication to go alongside my existing* [treatment] *and my levels have improved since so I think about it less and less … ’*

(PP24, 52 years old, FH mutation positive)

However, for some others notably with a VUS result, their outcome remained unclear and they had not emerged understanding if they had FH or not, or if there were possible implications for family members:

*‘Most of it* [seeing specialist] *was questions, questions … he didn’t explain … how I am ever going to find out whether I have got this condition or whether I ought to be telling my sons to be tested and what’s more important my new granddaughter to be tested.’*

(PP5, 62 years old, VUS)

Several patients with confirmed FH mutation had spoken to their relatives about testing after advice from the lipid specialist, but families subsequently experienced differing management. One patient with FH had contacted his siblings and daughter himself, and they had subsequently been referred by their GP for testing. In contrast, another was concerned that her son’s GP did not arrange referral for testing:

*‘I discussed it with my eldest who* [lives elsewhere] *… he did actually approach the doctor* [his GP] *and the doctor more or less laughed him out of the office and said, “Don’t worry, you’re too young to worry about things like that”* […] *so I am getting a bit concerned in case he does need this test.’*

(PP10, 49 years old, FH mutation positive, son 19 years old)

## DISCUSSION

### Summary

This study has found that introducing genetic testing with electronic case finding for FH into primary care was positively experienced by patients and practitioners. Although patients’ prior awareness of FH was low, their existing experience of cholesterol monitoring and treatment, or family histories, meant that they were unsurprised and positive about being offered testing having been identified as having a higher risk of FH. They saw this as helpful for themselves and to establish potential implications for family members, and found the process straightforward. However, those confirmed to have FH highlighted challenges for onward communication and testing in their families.

Healthcare professionals perceived this as an opportunity to enhance CVD prevention, similar to their existing screening for other disease. Noting some logistic challenges for required timing of blood sampling, they otherwise found electronic case finding and genetic testing were uncomplicated, and were open to further adoption of this approach in practice. They sought further guidance to better support detection and long-term care of FH, including clearly defined future clinical pathways with lipid specialists.

### Strengths and limitations

To the authors’ knowledge, this is the first study to investigate experience of introducing genetic testing with case finding for FH with patients from the general adult population identified as being at higher risk of FH by their general practice, and of practitioners using this approach in primary care. The sample was purposefully selected including patients from diverse social and educational backgrounds with a range of genetic test outcomes for FH, and healthcare professionals with different team roles from a range of practices. However, it should be noted that responders were self- selecting and findings must be interpreted with regard to the sample as described. Patients willing to be interviewed who did not take up the offer of testing were sought but could not be identified, as the vast majority of patients offered the opportunity accepted the testing.

Interviews were conducted by two researchers with backgrounds in social science, health services research, and health psychology, and analysis was developed jointly by both interviewers with a third senior clinical researcher, all from various disciplinary backgrounds. Steps to further enhance trustworthiness of analysis and check interpretation were also used, including member checking as described. Wider quantitative evaluation of the approach has recently been published.^11^

### Comparison with existing literature

This study adds to earlier work on detection of possible FH in general practice, as patient or practitioner experience has not been qualitatively reported, nor has previous research involved the use of genetic testing in primary care practice.^17^^–^23 The findings are consistent with wider research on what might help exploit genetics in the community setting such as use of family history,^24^ guidelines, and risk assessment tools.^25^ Reflecting on detection of FH, practitioner responders also highlighted opportunity to widen CVD prevention for patients and its similarities with established routine screening for other disease, such as diabetes. Practitioners sought more specific guidance on results and future management for FH, consistent with relative lack of understanding about this condition.^26^^,^^27^ It is noteworthy that examples of inappropriate advice to relatives of confirmed FH patients occurred in relation to cascade testing. Responders underlined the importance of having clear protocols that might be shared at the interface between primary care and lipid specialists to underpin effective identification and management of FH.

The current findings in primary care accord with previous research in specialist genetic and lipid settings with more selected patients (that is, patients already referred or being seen in a specialist setting rather than a general population) with FH or their relatives,^28^^–^31 where genetic testing for FH was well accepted and did not cause significant anxiety. Participants where an FH mutation was found in the current study mostly expected this. Most others felt relieved that they did not have the condition, although, for some, a negative result was more problematic or unsatisfactory, with lack of a genetic cause putting the onus for high cholesterol on the individual and their lifestyle. As found here, this may make people feel responsible and even guilty.^29^^,^^31^^,^^32^ As in previous research,^33^ the hereditary nature of FH and its impact for families was a further key concern for patient responders, including the need to protect children and grandchildren.^34^

### Implications for research and practice

Failure to detect FH leads to either no treatment or inadequate treatment of people wrongly thought to have more common ‘lifestyle-related’ elevated cholesterol. This study highlights patients’ and practitioners’ positive first experience of an approach with potential to help.^11^ FH is a current focus for policy to prevent avoidable premature disease and early deaths, and so is the only inherited disorder with a specific target to identify undiagnosed cases (25% of patients in the next 5 years) in the NHS Long Term Plan.^35^ Use of automated electronic case- finding tools such as FAMCAT in the current study,^10^^,^^11^ or others,^36^ could help, and exploit transferable skills in secondary prevention in primary care that are already well established, for example, in diabetes.

Raising public and health professional awareness of FH is needed. Should wider genetic testing in primary care be developed,^11^ then avoiding delays in receiving results or specialist assessment will be required. Patients may expect genetic testing to detect a mutation and provide an explanation for their elevated cholesterol because of their personal or family histories of this or of heart disease. Before testing, it may be helpful for patients to be advised that a negative result is the most common outcome. Practitioners should check understanding and be prepared to acknowledge a variety of patient responses, mostly of relief but also including surprise, guilt, or disappointment when FH is not identified. Practitioners should clarify that, even when a genetic cause (with implications for family members) is not found, addressing lifestyle causes and the need for treatment remain pertinent.

As genomic testing becomes more available, future research might also explore if still being found more prone to high cholesterol (polygenic hypercholesterolaemia) may help people make more sense of their own experience and family history. Finally, challenges and support for patients in communication of their results to wider family should be anticipated. In particular, clear processes to facilitate and ensure coordinated further testing for relatives of those with genetically confirmed FH are needed across service pathways.

Further research, including substantive randomised trial of this approach, is anticipated from parallel work.^11^ This is needed to assess whether detection of FH can be improved in primary care, including whether introducing genetic testing beyond its traditionally specialised arena into the community could enhance more targeted specialist referral and uptake, and widen access to, and yield more timely, diagnosis to improve outcomes. This should include assessment of intervention acceptability^37^ and requirements for implementation^38^ before its potential adoption in practice. Development of meaning or explanatory insights from experience of this approach could also be anticipated within future qualitative work.

This study has found introducing genetic testing with electronic case finding for FH into primary care was positively experienced by patients and practitioners. Further development of this approach could help improve detection of FH in the general population.
